# A sound children's mind in a healthy children's body

**DOI:** 10.3389/fnins.2014.00143

**Published:** 2014-06-10

**Authors:** Marco Taubert, Burkhard Pleger

**Affiliations:** ^1^Department of Neurology, Max Planck Institute for Human Cognitive and Brain SciencesLeipzig, Germany; ^2^Clinic for Cognitive Neurology, University Hospital LeipzigLeipzig, Germany

**Keywords:** children, physical fitness, functional magnetic resonance imaging (fMRI), cognitive control, prefrontal cortex, brain maturation

“Mens sana in corpore sano” (i.e., “A sound mind in a healthy body”) is possibly one of the phrases in human history with the widest range in meanings. Originally this phrase comes from *Satire X* of the Roman poet Juvenal (~60–127 AD). Juvenal's intention with this phrase was rather to teach his fellow Roman citizens the right virtues worth praying for, than making any scientific statement about the impact of physical activity on the human mind. But Juvenal's phrase interpreted in the latter context has turned true in light of growing evidence from neurocognitive research suggesting that physical fitness indeed enhances cognitive function (for a review see Hillman et al., 2008).

A more recent but even more important question is how physical activity and training may influence the developing brain during periods in which the brain matures, learns and forms connections (Amso and Casey, 2006). In adult animals and humans, physical exercise was in fact shown to influence the brains cellular and molecular layout by stimulating neurogenesis (e.g., transiently elevating levels of brain-derived neurotrophic factor important for brain plasticity and behavior) in brain regions like the hippocampus, known to underpin memory formation (for a review see Voss et al., 2013). Research like this in children is fundamental for the development of appropriate educational concepts optimally embedding sport activities into children's school day. Reality however speaks a different language, since even in light of previous research indeed suggesting improved cognitive function with enhanced physical fitness (see e.g., Hillman et al., 2009; Pontifex et al., 2011; Voss et al., 2011; Chaddock et al., 2012), opportunities for physical activities for children are progressively reduced, not only in school but also around their home environment (Troiano et al., 2008). At the same time, society is seeking for young sport talents in soccer, football, basketball, hockey, and other famous sports, progressively establishing a selection process segregating children into an inactive group, receiving more school class and less sport education, from those talents being intensively trained for a possible professional sport career.

That this development in sport education might be misleading was already suggested by a number of recent cross-sectional studies showing improved cognitive function with enhanced physical fitness (see e.g., Hillman et al., 2009; Pontifex et al., 2011; Voss et al., 2011; Chaddock et al., 2012). The study “The effect of physical activity on functional MRI activation associated with cognitive control in children: a randomized controlled intervention” by Laura Chaddock-Heyman and colleagues has gained a large community-wide interest after publication, since it is the first long-term interventional functional MRI study addressing the open question how physical activity influences cognitive capacity together with the function of specific prefrontal regions in children. The study published in Frontiers in Human Neuroscience in March 2013 is the consequent next step in this research field, since the cross-sectional studies conducted so far bear the risk that instead of the physical training itself, other hidden systematic between-group factors, such as genes and nutrition, may explain why physically active children outperformed their lower fit peers on tasks of cognitive control (Hillman et al., 2009; Pontifex et al., 2011; Voss et al., 2011; Chaddock et al., 2012). Furthermore, only a few studies on children have combined cognitive testing with brain imaging to examine how physical activity and aerobic fitness relate to brain processes of enhanced cognitive function (Hillman et al., 2011). Recent evidence from the few brain imaging studies published so far suggest reduced neural activity in the prefrontal cortex together with improved cognitive function in children with higher aerobic fitness levels (Chaddock et al., 2011; Voss et al., 2011). These findings are in agreement with studies comparing adult's with children's cognitive control capacities demonstrating reduced activity in the frontal cortex of adults coupled with enhanced cognitive performance (see e.g., Diamond, 2006; Bunge and Crone, 2009).

The study design used by Chaddock-Heyman and colleagues acknowledges these previous studies also when accounting for the two main problems that occur when investigating the developing brain: First, an interventional study involving children over a longer time period needs to control for the “normal” progress in brain maturation over the same period. Secondly, the interpretation of the findings requires a reference or a standard the acquired data can be compared with. To account for these problems, Chaddock-Heyman and colleagues selected a study design that allowed comparing the intervention group with two control groups, each addressing one of the two problems. The intervention group was investigated with functional magnetic resonance imaging on a Go/NoGo task before and after a 9 month period of physical activity (i.e., 60 min daily on 5 days per week). To control for the “normal” progress in brain maturation they recruited a control group in the same age range and with comparable educational background that were promised the participation in the same fitness program in future (i.e., so-called “wait-list group”). These subjects also underwent fMRI before and after a 9-month period but without participating in any fitness program in between. To address the second problem, namely establishing a reference the findings can be compared with, Chaddock-Heyman and colleagues recruited another control group consisting of college-aged young adults, since adult cognitive capacity together with the related brain function is often characterized as the “mature” or “optimal” model of brain function (Luna et al., 2010).

The study by Chaddock-Heyman and colleagues indicates that after the 9 months of physical training children of the intervention group indeed showed a decreased neural activity in the right anterior prefrontal cortex coupled with improvements in attentional and interference control. These differences in the pre-post comparison cannot be explained by “normal” brain maturation since children of the “wait-list group” did not show any changes neither in brain function, nor in cognitive control. Furthermore, when compared to the reference group consisting of colleague-aged adults, researchers found that children's behavior in the intervention group and brain activation pattern became more comparable to this reference cohort as compared to the “wait-list group.”

The findings by Chaddock-Heyman and colleagues demand wide public awareness since they are important with respect to children's health. Physical activity at young age is important in many perspectives, not only to prevent obesity and related diseases, which became a world-leading health burden over the last decades with dramatically climbing incidence rates especially in children, but also for optimally supporting the development of cognitive function and brain maturation. Children and their parents need to learn that being physically active is not only important for their health but also supports their cognitive development and hence enriches their life. Our modern society urgently needs to rethink the misleading development in physical education that should rather encourage each single child to become and stay active, instead of making sport accessible only for talents with possible perspectives in professional sports.

## Conflict of interest statement

The authors declare that the research was conducted in the absence of any commercial or financial relationships that could be construed as a potential conflict of interest.
